# Association between fulfilling the recommendations for health-enhancing physical activity with (instrumental) activities of daily living in older Austrians

**DOI:** 10.1007/s00508-019-1511-8

**Published:** 2019-05-22

**Authors:** Richard Crevenna, Thomas E. Dorner

**Affiliations:** 1 Department of Physical Medicine, Rehabilitation and Occupational Medicine, Medical University of Vienna, Vienna, Austria; 2 Unit Lifestyle & Prevention, Department for Social- und Preventive Medicine, Centre for Public Health, Medical University of Vienna, Kinderspitalgasse 15/I, 1090 Vienna, Austria

**Keywords:** Recommendation, Exercise, Activities of daily living (ADL), Instrumental activities of daily living (IADL), Resistance training, Endurance training

## Abstract

**Aim:**

The aim of the study was to describe the association between fulfilling the recommendation for health-enhancing physical activity (PA), and deficits in activities of daily living (ADL) and instrumental activities of daily living (IADL) in 3308 subjects aged 65+ years from the Austrian Health Interview Survey 2014.

**Methods:**

The proportion of subjects who fulfilled the minimal recommendations for health-enhancing PA was assessed with the Physical Activity Questionnaire of the European Health Interview Survey (EHIS-PAQ). The ADLs were assessed based on the Barthel index, and IADLs by the IADL scale of Lawton and Brody. Additionally, various sociodemographic and health-related factors were assessed.

**Results:**

Of the participants 54.7% did not fulfil the minimal requirements for aerobic PA, and 67.1% not for muscle strengthening PA, 16.4% reported ADL deficits, and 47.1% IADL deficits. Adjusted for sociodemographic and health-related parameters, not fulfilling the recommendations for aerobe PA was associated with a higher chance for ADL deficits (odds ratio, OR 1.73, 95%-confidence interval 1.36–2.21), and IADL deficits (1.57; 1.34–1.84). Not fulfilling the recommendations for muscle strengthening PA also increased the chance for ADL and IADLs deficits (1.34; 1.04–1.72, and 1.29; 1.09–1.53, respectively).

**Conclusion:**

The number of participants who did not fulfil the minimal requirements for aerobic or strengthening PA was very high, and these participants showed significantly more problems in ADLs and IADLs. Therefore, all future efforts should focus on increasing participation and adherence in exercise programs for older people with the intention to improve their performance status and functions in daily life.

## Introduction

Physical activity (PA) is one of the key determinants of physical, mental, and social health of children, adolescents but also of adults and older people. Health enhancing PA is associated with better health outcomes in all age groups, but especially in older people. Early development of health-relevant behavior is relevant [1]. Regular aerobic as well as muscle strengthening activities have been shown to be effective in improving performance status, functional status and quality of life [1]. Aerobic exercise has been shown to improve endurance capacity. Additionally, strength exercise in older people has been shown to increase muscular strength, physical performance, and partly muscle mass, and decrease frailty [2]. A recent correlation study of data from 11 European countries revealed a significant negative correlation between the proportion of people fulfilling the minimal aerobic physical activity recommendations (≥150 min/week) and the proportion of prefrail or frail people [3]. Exercise with the intention to improve flexibility of joints and to improve sensorimotor functions is also important for physical performance and quality of life. Regular PA including exercises targeting sensorimotor functions and balance, gait and muscle strength has been shown to prevent falls in older people living in the community [4]. Furthermore, cognitive frailty has been shown to be markedly associated with increased mortality in inactive older adults and being active has been shown to reduce the mortality risk among cognitively frail individuals. Therefore, engaging in physical activity seems to improve survival among cognitively frail older adults [5].

The Physical Activity Guidelines for Americans, 2nd edition are recommendations which provide information and guidance on the types and amounts of physical activity to improve a variety of health outcomes for multiple population groups: “Adults should do at least 150 minutes to 300 minutes a week of moderate-intensity, or 75 minutes to 150 minutes a week of vigorous-intensity aerobic physical activity, or an equivalent combination of moderate- and vigorous-intensity aerobic activity. They should also do muscle-strengthening activities on 2 or more days a week. Older adults should do multicomponent physical activity that includes balance training as well as aerobic and muscle-strengthening activities.” [6].

Evaluating activities of daily living (ADL) as well as instrumental activities of daily living (IADL) to describe health and function in older persons is part of the Austrian Geriatric Basic Assessment (Österreichisches Geriatrisches Basis-Assessment der ÖGGG, [7]). The ADL are often used as predictors of health and function in older persons. The European Network for Action on Ageing and Physical Activity recommends several measures such as the Barthel index to assess ADL in research and care practice in older populations [8]. The ability to maintain functional status has been shown to be an essential aspect of self-care for older adults. The IADLs are often overlooked, underassessed, and underreported, and the Lawton and Brody scale to measure IADLs has been described to be an ideal standard practice [9]. The number of people ≥65 years is increasing and the association of PA with ADL and with IADL seems to be relevant in planning future PA interventions with the goal of improving daily life in old people. It was the aim of the study to evaluate the association between fulfilling the recommendation for health-enhancing PA and impaired ADL and impaired IADL in subjects aged 65 years and older of the general community. Furthermore, it was the aim to examine if this association is mediated by various sociodemographic and clinical factors.

### Methods

The database for this analysis was the Austrian Health Interview Survey (ATHIS) 2014, a cross-sectional study based on the European Health Interview Survey (EHIS, [10–12]). In the survey, in 17 European countries, data on health status, health determinants, health care utilization, and sociodemographic and socioeconomic background data were collected.

In Austria, the survey was carried out by Statistics Austria from October 2013 to June 2015 via computer-assisted telephone interviewing (CATI). Some questions, including the questions on physical activity, were excluded from the CATI survey and participants were asked to fill in a paper questionnaire and return it via mail. For each Austrian NUTS-3 regions a sample size of 462 subjects (Viennese regions: 560 subjects) was aimed at, yielding in a gross sample size of 38,768 subjects. A total of 15,771 subjects were included in the survey (response rate: 40.7%). The response rate for the paper-based questionnaire was 93% [13]. For this analysis, weighted (according to geographic region, age, sex, family situation, migration background, and education level) data from 3398 subjects aged 65 years and older were used. In this survey, missing values were imputated [13].

To assess the proportion of subjects who fulfilled the minimal recommendations for health-enhancing PA, the Physical Activity Questionnaire of the EHIS (EHIS-PAQ) was used [14]. Here, only activities lasting for longer than 10 min were considered. For aerobic PA, the minutes spent in a typical week with cycling, doing sports, fitness, and being at least moderately physically active in the leisure time were added and dichotomized as being active at least 150 min per week or not. For muscle strengthening PA subjects were asked whether they performed muscle strengthening activities in a typical week at all, and if yes, on how many days. Again, the variable was dichotomized as doing muscle strengthening activities at least twice a week or not.

The following sociodemographic data were used: participants’ sex (male or female) and age in 5‑year intervals; living conditions were evaluated in two categories, living with a partner vs. living alone; education level was obtained in three levels, primary education (compulsory school), secondary education (apprenticeship school, professional/commercial school and high school) and tertiary education (university).

The following clinical parameters were used: body mass index (BMI) category, based on self-reported data for body weight and body height, and categorized as underweight (BMI <18.5 kg/m^2^), normal weight (BMI 18.5–24.9 kg/m^2^), overweight (25.0–29.9 kg/m^2^) and obesity (BMI ≥30 kg/m^2^). Furthermore, participants were asked whether they suffered from any of 17 listed chronic diseases within the last 12 months or not.

The ADLs were assessed based on the Barthel Index [15] and the following ADLs were evaluated: eating and drinking, getting up and sitting down on a chair or on a bed, dressing, using the toilet, and showering or bathing. The IADLs were assessed based on a scale proposed by Lawton and Brody [16] and the following IADLs were evaluated: preparing meals, using the telephone, shopping, organizing medication, doing light housework, doing occasionally heavy housework, and managing financial and administrative matters. For each ADL the persons could choose between the following categories: “no difficulties”, “some difficulties”, “major difficulties”, and “I am not able to do that”. For each IADL subjects could choose between the same categories and additionally the category “not applicable”. For the analysis, ADLs and IADLs were dichotomized as having no problem at all in any ADL or IADL vs. having at least some difficulties in at least one ADL or IADL. In a sensitivity analyses the ADLs and IADLs were dichotomized as having no or some difficulties in the ADLs of IADLs, vs. having at least major difficulties in at least one ADL or IADL.

For descriptive statistics, categorical variables are presented as number of subjects in each category and percentages. In stepwise binary logistic regression analyses the association between fulfilling the aerobic or muscle strengthening recommendations for PA with problems in ADLs or IADLs (dependent variables) was assessed. The first model is unadjusted, the second model was adjusted for sex, age, living condition, and education level, and the third model was additionally adjusted for BMI category and presence of chronic conditions. All parameters except age were used as categorical variables. Results are presented as odds ratios (OR) and 95% confidence intervals (95% CI), and also the estimates for the co-variables are presented. Calculations were performed using SPSS Statistics 22.

### Results

In Table 1 the sociodemographic, clinical and PA-related characteristics are presented. About half of the participants did not fulfil the minimum requirements for aerobic PA, and two thirds did not fulfil the minimum requirements for muscle strengthening PA. About one third were living alone, and the most common category for education was secondary education level. Most subjects were overweight or obese. The most common chronic disease was hypertension (which affected about half of the subjects), followed by chronic back pain and osteoarthritis (which affected about one third of the subjects). About one fifth of the participants had problems in ADLs, and about half of the participants had problems in IADLs.

**Table 1:** Sociodemographic, clinical and exercise-related characteristics of 3308 subjects in the general Austrian community aged 65 years and older

		*N*	%
Exercise related factors	Not performing at least 150 min/week aerobic exercise	1809	54.7
	Not performing at least twice/week strength training	2220	67.1
Sex	Male	1438	43.5
	Female	1870	56.5
Living conditions	Living alone	1299	39.3
	Living with partner	2009	60.7
Education level	Primary education	1171	35.4
	Secondary education	1586	47.9
	Tertiary education	551	16.6
Body mass index category	Underweight	59	1.8
	Normal weight	1238	37.4
	Overweight	1358	41.0
	Obesity	649	19.6
Chronic diseases	Bronchial asthma	206	6.2
	Chronic obstructive pulmonary disease	286	8.6
	Heart attack or chronic condition after heart attack	97	2.9
	Coronary heart diseases or angina pectoris	228	6.9
	Hypertension	1598	48.3
	Stroke or chronic condition after stroke	70	2.1
	Osteoarthritis	1020	30.8
	Diabetes mellitus	439	13.3
	Chronic back pain	1261	38.1
	Chronic neck pain	837	25.3
	Allergy (coryza, food, dermatitis)	561	17.0
	Hepatic cirrhosis	10	0.3
	Urinary incontinence	417	12.6
	Kidney disease or renal insufficiency	136	4.1
	Depression	368	11.1
	Chronic headache	155	4.7
	Gastric or duodenal ulcers	86	2.6
ADL/IADL deficits	ADL deficits	544	16.4
	IADL deficits	1558	47.1

*ADL* activities of daily living, *IADL* instrumental activities of daily lifing

Not fulfilling the recommendations for aerobic PA tripled the chance for ADL deficits and doubled the chance for IADL deficits (unadjusted model). Adjusting for sociodemographic factors diminished the OR only slightly. Age, living alone, and having low education were significantly associated with problems in ADLs and IADLs. Additionally, adjusting for clinical factors clearly diminished the OR, but not fulfilling the recommendations for aerobe PA sill significantly increased the chance for ADL deficits by 70% and for IADL deficits by 60% (fully adjusted model). Overweight, obesity, chronic obstructive pulmonary disease (COPD), heart attack or chronic condition after heart attack, stroke or chronic condition after stroke, osteoarthritis, chronic back pain, chronic neck pain, urinary incontinence, kidney disease or renal insufficiency, depression, and chronic headache were significantly associated with problems in ADLs, and most of them also with IADLs (Table 2).

**Table 2:** Association between not fulfilling minimum requirements for aerobic health-enhancing physical activity and presence of ADL deficits and IADL deficits stepwise adjusted for possible confounders

	ADL deficits						IADL deficits					
	Model I		Model II		Model III		Model I		Model II		Model III	
	OR	95% CI	OR	95% CI	OR	95% CI	OR	95% CI	OR	95% CI	OR	95% CI
Not performing at least 150 min/week aerobic exercise	2.92	2.37–3.59	2.43	1.96–3.01	1.73	1.36–2.21	2.14	1.86–2.46	1.87	1.62–2.17	1.57	1.34–1.84
Sex (ref: female)	–	–	1.02	0.82–1.28	1.34	1.03–1.74	–	–	1.08	0.92–1.27	1.22	1.03–1.46
Age (5 years intervals)	–	–	1.36	1.27–1.47	1.50	1.38–1.64	–	–	1.44	1.36–1.52	1.48	1.39–1.58
Living alone (ref: living with partner)	–	–	1.68	1.35–2.09	1.65	1.30–2.11	–	–	1.05	0.89–1.23	0.92	0.77–1.10
Primary education (ref: tertiary)	–	–	1.76	1.30–2.40	1.48	1.04–2.11	–	–	1.83	1.46–2.29	1.60	1.25–2.04
Secondary education (ref: tertiary)	–	–	1.08	0.80–1.48	1.20	0.85–1.69	–	–	1.59	1.29–1.96	1.68	1.34–2.11
Underweight (ref: normal weight)	–	–	–	–	0.21	0.08–0.57	–	–	–	–	1.31	0.70–2.47
Overweight (ref: normal weight)	–	–	–	–	1.31	1.00–1.71	–	–	–	–	0.90	0.76–1.08
Obesity (ref: normal weight)	–	–	–	–	2.54	1.87–3.44	–	–	–	–	1.93	1.43–2.62
Bronchial asthma	–	–	–	–	0.86	0.55–1.35	–	–	–	–	0.92	0.65–1.29
Chronic obstructive pulmonary disease	–	–	–	–	1.44	1.01–2.05	–	–	–	–	1.93	1.43–2.62
Heart attack or chronic condition after heart attack	–	–	–	–	2.00	1.14–3.52	–	–	–	–	1.48	0.86–2.54
Coronary heart diseases or angina pectoris	–	–	–	–	1.33	0.91–1.96	–	–	–	–	1.33	0.95–1.87
Hypertension	–	–	–	–	0.79	0.63–1.00	–	–	–	–	0.85	0.72–0.99
Stroke or chronic condition after stroke	–	–	–	–	5.10	2.73–9.51	–	–	–	–	3.32	1.77–6.24
Osteoarthritis	–	–	–	–	2.49	1.96–3.16	–	–	–	–	1.58	1.32–1.90
Diabetes mellitus	–	–	–	–	1.12	0.82–1.52	–	–	–	–	1.07	0.84–1.36
Chronic back pain	–	–	–	–	2.01	1.57–2.57	–	–	–	–	2.17	1.82–2.59
Chronic neck pain	–	–	–	–	1.43	1.11–1.85	–	–	–	–	0.88	0.72–1.07
Allergy (coryza, food, dermatitis)	–	–	–	–	1.04	0.77–1.39	–	–	–	–	1.39	1.12–1.71
Hepatic cirrhosis	–	–	–	–	1.24	0.20–7.57	–	–	–	–	0.53	0.13–2.21
Urinary incontinence	–	–	–	–	1.50	1.13–2.00	–	–	–	–	2.01	1.55–2.60
Kidney disease or renal insufficiency	–	–	–	–	2.55	1.64–3.96	–	–	–	–	1.37	0.89–2.12
Depression	–	–	–	–	2.73	2.05–3.63	–	–	–	–	2.22	1.69–2.93
Chronic headache	–	–	–	–	1.69	1.07–2.66	–	–	–	–	2.76	1.78–4.29
Gastric or duodenal ulcers	–	–	–	–	1.07	0.54–2.14	–	–	–	–	0.91	0.54–1.54

Model I: unadjusted; Model II: adjusted for socio-demographic parameters; Model III: additionally adjusted for health-related parameters

*ADL* activities of daily living; *IADL* instrumental activities of daily living; *OR* odds ratio; *CI* confidence interval

Not fulfilling the recommendations for muscle strengthening PA increased the chance for deficits in ADLs by 50% and for IADLs by 40% (unadjusted). Again, sociodemographic and clinical factors diminished the ORs, and in the fully adjusted model, not fulfilling the recommendations for muscle strengthening PA increased the chance for ADL and IADLs deficits significantly by 30% (Table 3).

**Table 3:** Association between not fulfilling minimum requirements for strength training and presence of ADL deficits and IADL deficits stepwise adjusted for possible confounders

	ADL deficits						IADL deficits					
	Model I		Model II		Model III		Model I		Model II		Model III	
	OR	95% CI	OR	95% CI	OR	95% CI	OR	95% CI	OR	95% CI	OR	95% CI
Not performing at least twice/week strength training	1.53	1.24–1.88	1.54	1.24–1.91	1.34	1.04–1.72	1.38	1.19–1.59	1.36	1.17–1.59	1.29	1.09–1.53
Sex (ref: female)	–	–	0.98	0.79–1.23	1.31	1.01–1.70	–	–	1.04	0.89–1.22	1.20	1.00–1.43
Age (5 years intervals)	–	–	1.41	1.31–1.52	1.53	1.40–1.68	–	–	1.47	1.39–1.56	1.50	1.41–1.60
Living alone (ref: living with partner)	–	–	1.73	1.39–2.15	1.67	1.31–2.13	–	–	1.07	0.91–1.25	0.94	0.79–1.12
Primary education (ref: tertiary)	–	–	1.79	1.32–2.43	1.48	1.04–2.10	–	–	1.87	1.50–2.34	1.61	1.26–2.05
Secondary education (ref: tertiary)	–	–	1.07	0.79–1.46	1.17	0.83–1.66	–	–	1.57	1.28–1.94	1.67	1.33–2.10
Underweight (ref: normal weight)	–	–	–	–	0.23	0.09–0.62	–	–	–	–	1.25	0.67–2.34
Overweight (ref: normal weight)	–	–	–	–	1.25	0.96–1.63	–	–	–	–	0.89	0.75–1.06
Obesity (ref: normal weight)	–	–	–	–	2.56	1.87–3.45	–	–	–	–	1.22	0.98–1.53
Bronchial asthma	–	–	–	–	0.88	0.56–1.38	–	–	–	–	0.92	0.65–1.30
Chronic obstructive pulmonary disease	–	–	–	–	1.45	1.02–2.06	–	–	–	–	1.97	1.46–2.67
Heart attack or chronic condition after heart attack	–	–	–	–	1.95	1.11–3.43	–	–	–	–	1.45	0.85–2.50
Coronary heart diseases or angina pectoris	–	–	–	–	1.38	0.94–2.03	–	–	–	–	1.40	1.00–1.96
Hypertension	–	–	–	–	0.80	0.63–1.00	–	–	–	–	0.85	0.72–0.99
Stroke or chronic condition after stroke	–	–	–	–	5.11	2.74–9.54	–	–	–	–	3.39	1.80–6.37
Osteoarthritis	–	–	–	–	2.59	2.04–3.28	–	–	–	–	1.62	1.35–1.94
Diabetes mellitus	–	–	–	–	1.15	0.84–1.57	–	–	–	–	1.09	0.86–1.39
Chronic back pain	–	–	–	–	2.04	1.60–2.61	–	–	–	–	2.21	1.85–2.64
Chronic neck pain	–	–	–	–	1.43	1.11–1.85	–	–	–	–	0.88	0.72–1.07
Allergy (coryza, food, dermatitis)	–	–	–	–	1.01	0.76–1.35	–	–	–	–	1.34	1.09–1.65
Hepatic cirrhosis	–	–	–	–	1.17	0.18–7.50	–	–	–	–	0.52	0.12–2.23
Urinary incontinence	–	–	–	–	1.56	1.17–2.07	–	–	–	–	2.02	1.56–2.61
Kidney disease or renal insufficiency	–	–	–	–	2.56	2.08–3.68	–	–	–	–	1.38	0.90–2.13
Depression	–	–	–	–	2.77	2.08–3.68	–	–	–	–	2.26	1.71–2.99
Chronic headache	–	–	–	–	1.71	1.09–2.68	–	–	–	–	2.78	1.79–4.31
Gastric or duodenal ulcers	–	–	–	–	1.03	0.52–2.05	–	–	–	–	0.90	0.53–1.53

Model I: unadjusted; Model II: adjusted for socio-demographic parameters; Model III: additionally adjusted for health-related parameters

*ADL* activities of daily living; *IADL* instrumental activities of daily living; *OR* odds ratio; *CI* confidence interval

The sensitivity analysis with the alternative categorization of being affected from deficits in ADLs and IADLs did not change the reported associations. Of the subjects 148 (4.5%) were affected from severe ADL deficits, and 1230 subjects (37.2%) from severe IADL deficits. The respective OR (95% CI) in the fully adjusted models regarding fulfilling the minimum requirements for the aerobic criteria for PA were 4.04 (2.27–7.20) for deficits in ADLs and 1.82 (1.55–2.14) for deficits in IADLs, and regarding fulfilling the minimum criteria for muscle strengthening PA were 2.07 (1.26–3.40) for deficits in ADLs and 1.25 (1.05–1.48) for deficits in IADLs.

### Discussion

Although regular PA has proven effects for performance status, health, functional status and quality of life of older people, 54.7% of the Austrian participants aged 65 years and over did not fulfil the minimum requirements for aerobic PA, and 67.1% of the participants did not fulfil the minimum requirements for muscle strengthening PA. Of the participants 83.3% showed a primary or secondary education level, and 39.3% were living alone. Most of them (60.6%) were overweight or obese and showed typical chronic diseases indicating a sedentary life style, such as hypertension (48.3%), chronic back pain (38.1%) and osteoarthritis (30.8%) 16.4% reported ADL deficits, and 47.1% IADL deficits. The present results are in line with the recent literature. The proportion of persistently physically inactive individuals has been shown to be relatively high at all ages [17]. Nevertheless, the proportion of persistently physically inactive individuals seems to increase with age. Persistent or increasing PA has been described to be associated with male gender, being Caucasian, non-smoking, having low television viewing time and higher socioeconomic status, no chronic illnesses, and family support for PA [18].

Persistently physically inactive individuals seem to show significantly more problems in ADLs and IADLs as shown in the present study. Not fulfilling the recommendations for aerobic PA significantly increased the chance for ADL deficits by 70% and for IADL deficits by 60%. Not fulfilling the recommendations for muscle strengthening PA increased the chance for ADL and IADLs deficits significantly by 30%. The number of participants who did not fulfil the minimum requirements for aerobic and/or strengthening PA was high, and these participants seemed to show significantly more problems in ADLs and IADLs. Therefore, all future efforts should focus on increasing participation and adherence in exercise programs for older people with the intention to improve their performance status and functions in daily life. Exercise is known to be able to reduce the risk of disability for ADL. This has been shown in Austrian studies, as well as in systematic reviews and meta-analyses [19–21]. In an 8‑year population-based prospective cohort study in 1003 community-dwelling older Japanese women without ADL disability in the baseline surveys the examination of longitudinal associations between 16 different exercise types and the incidence of ADL disability revealed that especially dancing seems to contribute to a reduced risk of ADL disability in older women [22]. Exercise is also known to delay the decline of IADL. In a 4-year prospective cohort study to examine the longitudinal associations between exercise types and the onset of IADL decline in older women, the authors were able to show that participation in calisthenics seemed to be significantly and independently associated with delayed IADL decline in older women aged ≥75 years [23]. Furthermore, PA is associated with better health outcomes in older people. Therefore, all efforts should focus on increasing participation and adherence in exercise programs for older people with the intention to improve their performance status and quality of life. The measurement of health effects in lay volunteers who made home visits consisting of social interaction, nutritional and physical exercise interventions to pre-frail and frail older people revealed that such projects seem to have additional limited health benefits [24].

Intrapersonal motivators, such as health benefits have been shown to be the most common motivators to participate in a physical exercise or nutritional program. Intrinsic health beliefs, fear of falling or injuries, influence of significant others and the environment, and (para)medical encouragement have been identified to be concepts to participate in a physical exercise intervention [25].

Adjusting for sociodemographic parameters (model II in the logistic analysis), and especially adjusting for health-related parameters (model III) lowered the odds ratios of exercise related factors towards ADL and IADL deficits. That means that socio-demographic and health related parameters partly mediated the association between exercise and ADL/IADL deficits. In this analysis, especially higher age, and lower education were associated with ADL/IADL deficits, which was in the light of existing scientific literature not unexpected [26]. Regarding health-related parameters, obesity, but not overweight was associated with a higher risk of ADL/IADL deficits. This is in line with previous findings, where in subjects aged 65 years and older, obesity, but not overweight was associated with adverse health outcomes [27, 28]. Regarding chronic diseases, especially stroke has to be mentioned as the chronic disease which was most strongly associated with ADL/IADL deficits. This can be explained in the way that stroke often leads to motoric and sensory deficits which deteriorate ADL [29, 30] but also musculoskeletal disorders, such as chronic back pain and osteoarthritis were clearly associated ADL/IADL deficits, which is also in line with previous findings [31].

A strength of this study is that the analyzed sample was taken from the general population aged 65 years and over and not from the clinical setting. Additionally, the large and representative sample as well as using standardized methods to measure PA, ADL and IADL have to be mentioned as strengths. Potential limitations include that the measures were all self-reported and not objectively measured. Furthermore, the cross-sectional design of the study should be mentioned, which shows a clear and robust association between amount of PA and deficits in ADL and IADL but does not allow conclusions to be drawn regarding direction of the association and timeline between exposure and outcome.

In conclusion, this cross-sectional study using the ATHIS database 2014 revealed an enormous number of Austrian participants who did not fulfil the minimum requirements for aerobic and/or strengthening PA. Furthermore, these participants not fulfilling the minimum requirements seemed to have significantly more problems in ADLs and IADLs. Therefore, all future efforts for preventive measures should focus on increasing participation and adherence in exercise programs for older people of the community with the intention to improve their performance status and functions in daily life, ADL and IADL. In our opinion, these strategies have to start in very early childhood and have to last through all different age phases until senior (great) age.
